# PrEP awareness and protective barrier negotiation among transgender people attracted to men in Aotearoa New Zealand

**DOI:** 10.1002/jia2.25980

**Published:** 2022-10-12

**Authors:** Jack L. Byrne, Kyle K. H. Tan, Peter J. Saxton, Ryan M. Bentham, Jaimie F. Veale

**Affiliations:** ^1^ Trans Health Research Lab School of Psychology University of Waikato Hamilton New Zealand; ^2^ Faculty of Māori and Indigenous Studies University of Waikato Hamilton New Zealand; ^3^ School of Population Health Faculty of Medical and Health Sciences University of Auckland Auckland New Zealand; ^4^ AIDS Epidemiology Group Department of Preventive and Social Medicine University of Otago Dunedin New Zealand

**Keywords:** transgender, sexuality, condom, pre‐exposure prophylaxis, HIV, New Zealand

## Abstract

**Introduction:**

Internationally, trans women are disproportionately impacted by HIV, encounter specific barriers navigating safer sex and face inequities accessing HIV prevention, including pre‐exposure prophylaxis (PrEP). Aotearoa/New Zealand (hereafter Aotearoa) was one of the first countries internationally to publicly fund PrEP in 2018, including for trans people. However, few data exist on PrEP awareness or sexual negotiation among trans populations to guide implementation. We present the first Aotearoa data on trans people's ability to negotiate barrier protection and awareness of PrEP efficacy and availability.

**Methods:**

We used data from a large, diverse community‐based nationwide survey of trans (including non‐binary) people in Aotearoa: Counting Ourselves (*N* = 1178) conducted from 21 June to 30 September 2018. Generalized regression analyses were carried out among participants who have had sex (*n* = 704; M_age_ = 32.5) to identify associations between demographic factors (age, gender and sexual attraction, ethnicity, income, education qualification and current sex work involvement) and the Trans‐Specific Barrier Negotiation Self‐Efficacy (T‐Barrier) Scale and PrEP awareness.

**Results:**

The mean value of a 40‐point T‐Barrier Scale was 33.45 (SD: 6.89), suggesting a relatively high perceived ability among our participants to negotiate protective barrier usages in different situations. Asian participants scored 3.46 points lower compared to Pākehā (White) participants, and trans women attracted to men (cisgender and/or trans men) scored 2.40 points higher than trans women not attracted to men. Three‐fifths (59.7%) were aware that PrEP reduced HIV risks and did not prevent sexually transmitted infections (STI) transmission, and only two‐fifths (40.2%) knew PrEP was publicly funded for trans people. In multivariate models, we found participants who were older, trans women or those with lower education qualifications were less likely to have increased levels of PrEP awareness.

**Conclusions:**

Participants attracted to men have a higher potential need for PrEP and were more likely to report PrEP awareness and that they could negotiate protective barrier usage. However, trans women and those with lower educational qualifications reported lower levels of PrEP awareness. More trans‐competent sexual health education, drawing on the newly released PrEP guidelines, is needed to promote the benefits of PrEP in the Aotearoa HIV epidemic context, particularly for trans women.

## 1 INTRODUCTION

Initial international data specific to transgender (trans) people demonstrate a heavy burden of HIV among trans women, specifically trans women who have sex with men [1]. HIV inequities persist for trans women, with some limited data now available about the specific vulnerabilities of non‐binary people and trans men. The first US national probability survey of trans people (TransPoP), conducted between 2016 and 2018, found HIV infection was highest among trans women (6.5%), followed by 5.1% for non‐binary people and 0.8% for trans men [2]. Overall, the US trans population had more than three times the odds of self‐reporting HIV infection compared to their cisgender counterparts (7.1% vs. 2.1%; odds ratio = 3.56) [2].

Trans people in Aotearoa/New Zealand (hereafter, Aotearoa) include trans women, trans men, people with non‐binary genders and those who identify with non‐Western gender diverse identities, including indigenous Māori terms whakawahine or tangata ira tāne, and the Samoan term fa'afafine [3]. In this article, we use the term “trans” to refer to people who identify their gender as different from their sex assigned at birth. The only population‐based study that has collected data on the size of the trans adult population in Aotearoa, the Household Economic Survey, found that 0.8% were trans [4].

Aotearoa has had a successful record controlling HIV, being one of the first countries to record a decline in AIDS diagnoses in the 1990s [5]. Annual per‐capita HIV diagnosis rates have remained low [6] and in 2019 were 2.4/100,000 adults [7]. The epidemic is concentrated in men who have sex with men (MSM) who comprised over three‐quarters of all locally acquired HIV diagnoses in 2019 [7]. HIV transmission among other key populations has been effectively contained, including sex workers [8] and people who inject drugs [9]. Internationally, Aotearoa has among the lowest HIV prevalence and incidence in these groups. This outcome is mainly due to progressive public health and human rights law reform, such as public funding of needle exchange programmes in 1988 and decriminalization of sex work in 2003. Since the initiation of enhanced epidemiological surveillance in 1996, few trans individuals have been recorded with an HIV diagnosis in Aotearoa (0.5%), with only 23 of 4323 recorded diagnoses between 1996 and 2020 [10]. Further, no evidence of hidden undiagnosed infection among trans people has been identified by sentinel surveillance at sexual health clinics [11]. However, this picture is likely to underestimate incidence due to incomplete reporting, misclassification or small sample sizes. For example, 1.7% of those identified with HIV in 2018 in Aotearoa were trans women [12], indicating transmission among trans people may be more common than previously thought. There is currently no population‐based estimate of HIV infection among trans people in Aotearoa.

Trans people in Aotearoa experience discrimination and a lack of relevant and culturally competent health services [3], resulting in high unmet health needs. Transpeople have tended to be excluded from HIV prevention research that has focused on cis MSM [8]. For example, behavioural surveillance has historically presented identity options for fa'afafine but no explicit option for trans men, trans women or non‐binary people [13]. Similarly, HIV prevention organizations have been funded to prioritize cis MSM with an unclear remit regarding trans communities. These practices contribute to invisibility surrounding trans people's experience of HIV prevention specifically and safe sex and sexual health more broadly. Our nationwide community survey found only 9% of trans people in Aotearoa have ever received any information about sexually transmitted infections (STI) prevention or safer sex that was specific to trans people [3].

Aotearoa was also one of the first countries to publicly fund pre‐exposure prophylaxis (PrEP) in March 2018 on a targeted basis [14]. Eligible individuals had to be male or trans and to have had sex with a male, as well as fulfilling other criteria, such as having engaged in receptive anal intercourse with casual partners, having been recently diagnosed with rectal gonorrhoea, rectal chlamydia or infectious syphilis, or having recently used methamphetamine. Based on these eligibility criteria [14], researchers estimated that 5847 individuals would be eligible for PrEP, noting that the lack of official statistics on trans adults precluded a reliable estimate [15]. Our study took place in June 2018 and provides baseline data on PrEP awareness. Further waves of the Counting Ourselves survey will explore links between awareness and the use of PrEP. Calls for greater flexibility in PrEP provision resulted in Aotearoa's first formal PrEP prescribing guidelines in 2021 [12] that included specific sections on trans individuals, to help physicians and trans individuals decide whether PrEP would be beneficial.

Aotearoa's epidemiological, policy and healthcare context presents a unique setting for examining the experiences of trans people concerning HIV. This is because while there are data showing some key populations (notably sex workers and people who inject drugs) have low rates of HIV infection in Aotearoa, trans people remain invisible in research and surveillance data here. We present the first Aotearoa data on trans people's ability to negotiate barrier protection and awareness of PrEP efficacy and availability.

## 2 METHODS

### 2.1 Procedure

We performed data analyses on the 2018 Counting Ourselves: Aotearoa New Zealand Trans and Non‐Binary Health Survey. As a trans‐led community‐based survey, Counting Ourselves aimed to counter the long‐held invisibility of trans people in national statistics by asking participants questions from national population‐based surveys (e.g. the New Zealand Health Survey [16]), overseas trans surveys (e.g. the US Trans Survey [17] and Trans PULSE Ontario [18, 19]), and questions designed in collaboration with the survey's community advisory group. Eligible participants met the following criteria: (1) aged 14 or above; (2) identified as trans or non‐binary; and (3) residing in Aotearoa. Participants were recruited through community networks and organizations, with community leaders fronting social media posts to harder‐to‐reach trans communities (including indigenous Māori, Pasifika, Asian and older people). In addition, we promoted the survey through networks of health professionals and academic researchers working in trans health.

The New Zealand Health and Disability Ethics Committee approved the study procedure (18/NTB/66/AM01). Participants were allowed sufficient time to read through the information sheet that outlined their rights and information on accessing support if the survey topics raised concerns or were stressful. Participants gave their consent by completing the survey. The survey was available to self‐complete online with the option of requesting a paper copy and pre‐paid envelope to return it. More details about the survey methods can be read in the published community report [3].

### 2.2 Measures

#### 2.2.1 Gender

Participants were categorized into four gender groups based on responses to two questions on sex assigned at birth and current gender identification. Trans men were those assigned female at birth (AFAB) who identified as a man, trans man, transsexual and/or as the indigenous Māori identity tangata ira tāne. Trans women were those assigned male at birth (AMAB) who identified as a woman, trans woman, transsexual, or as the indigenous Māori identities tangata ira wahine, and/or whakawahine, or using other culturally specific identities, such as the Samoan term fa'afafine. Participants who did not meet these criteria were classified either as nonbinary AFAB or nonbinary AMAB.

#### 2.2.2 Sexual attraction

Using the question from Trans PULSE Ontario [18, 19], participants were asked “Who are you sexually attracted to? Mark all that apply.” Response options included “trans men,” “cis men,” “trans women,” “cis women,” “genderqueer or non‐binary people,” “none of the above” and “others.” As HIV prevalence in Aoteaora is concentrated among MSM [7], we created a nominal variable “gender and sexual attraction” that differentiated between participants who are sexually attracted to men—either trans or cis men—and those who are not (Table 1). We chose trans women who are sexually attracted to men as the reference group given previous literature has identified this group as vulnerable to HIV risk [2, 7].

**Table 1:** Demographic details of Counting Ourselves participants who have ever had sex (*N* = 704)

	*n* (%)
Age groups	
14–18	51 (7.2)
19–24	192 (27.3)
25–39	283 (40.2)
40–54	111 (15.8)
55+	67 (9.5)
Gender groups	
Trans women	227 (32.3)
Trans men	185 (26.4)
Non‐binary people AFAB	218 (31.1)
Non‐binary people AMAB	72 (10.3)
Prioritized ethnicity groups	
Māori	90 (12.8)
Pasifika	20 (2.8)
Asian	25 (3.6)
Pākehā/New Zealand European (White)	549 (78.0)
Others including MELAA	20 (2.8)
Regions	
Auckland	217 (31.4)
Wellington	206 (29.8)
Other north island	116 (16.8)
Other north island	76 (11.0)
Other south island	76 (11.0)
Personal income in the last 12 months	
Loss and zero	42 (6.4)
1–15,000	202 (30.7)
15,001–50,000	257 (39.1)
50,001 and more	156 (23.7)
Education qualification	
None	30 (4.6)
Levels 1–5 (certificate)	293 (44.6)
Levels 6 and 7 (diploma and bachelor)	177 (26.9)
Level 8 and above (postgraduate)	157 (23.9)
Gender and sexual attraction^a^	
Trans women attracted to men	113 (16.2)
Trans women not attracted to men	112 (16.1)
Trans men attracted to men	145 (20.8)
Trans men not attracted to men	39 (5.6)
Non‐binary AFAB attracted to men	164 (23.3)
Non‐binary AFAB not attracted to men	52 (7.5)
Non‐binary AMAB attracted to men	53 (7.6)
Non‐binary AMAB not attracted to men	19 (2.7)
Ever engaged in sex work	135 (19.7)
Engaged in sex work in the past year	49 (7.2)

Abbreviations: AFAB, assigned female at birth; AMAB, assigned male at birth; MELAA, Middle Eastern/Latin/African.

^a^ There were two non‐binary participants who did not report sex assigned at birth.

#### 2.2.3 Ethnicity

Participants were asked the New Zealand Health Survey's ethnicity question which permits multiple responses. Using the Ministry of Health's ethnicity prioritization protocol [20], we classified participants into one of the four ethnic groups in the priority order of Māori, Pasifika, Asian and New Zealand European/Pākehā (approximately equivalent to White in other contexts) or other.

#### 2.2.4 T‐Barrier Scale

The T‐Barrier 8‐item Scale was adopted from Trans PULSE Ontario to assess participants’ perceived ability to negotiate protective barrier use in different situations with a sexual partner [18]. For example, participants were asked to rate their level of certainty on an ordinal scale from “not at all certain (1)” to “completely certain (5)” about using protection when meeting a new partner, a cisgender partner and a trans or non‐binary partner. Total scores of the T‐Barrier Scale range from 8 to 40. All eight items demonstrated high factor loading in a one‐factor construct which explained 65% of the variance in the Ontario sample [18]. Similar to previous studies [18, 21], the internal consistency of the T‐Barrier Scale in the current dataset was high (Cronbach's α = 0.92).

#### 2.2.5 PrEP awareness

Our research team created three questions about awareness of PrEP provision in Aotearoa (Table 2). Participants who responded “I wasn't sure” were treated as missing in the regression models.

**Table 2:** Percentage of participants who were aware of the following PrEP information (*N* = 685)

	I knew that *n* (%)	I wasn't sure *n* (%)	I didn't know that *n* (%)
1. PrEP (pre‐exposure prophylaxis) is a pill that, if taken every day by someone who is HIV negative, significantly decreases their risk of acquiring HIV	409 (59.7)	58 (8.5)	218 (31.8)
2. If taken correctly, PrEP significantly reduces the risk of acquiring HIV but it does not prevent the transmission of other STIs like gonorrhoea and syphilis^a^	407 (59.7)	48 (7.0)	227 (33.3)
3. PrEP is now publicly funded in New Zealand, if you are “male or transgender” and meet other eligibility criteria^a^	274 (40.2)	86 (12.6)	322 (47.2)

^a^ Compared to the first statement, there were three participants who did not respond to these statements out of participants who have ever had sex.

### 2.3 Data analysis

All statistical analyses were conducted in IBM SPSS Statistics version 27. Missing data for education qualification (2.6%) and income (13.1%) were imputed using the expectation maximization (EM) method based on means and covariances of related socio‐economic status measures, such as employment status and deprivation. We also imputed the missing data for participants who had responded to at least two items on the T‐Barrier Scale (ranging from 10.7% to 22.5%) using the EM method. The high percentage of missing values for T‐Barrier items included “this does not apply” responses (see Appendix S1).

Next, using Chi‐square goodness of fit tests, we determined whether the proportion of participants reported being somewhat or completely certain varied across gender and sexual attraction groups. Next, we undertook generalized linear regression analyses to examine characteristics associated with the T‐Barrier Scale, and logistic regressions for each of the three PrEP awareness statements. Variables that displayed statistically significant differences in bivariate models were treated as covariates in multivariate models. In all analyses, an alpha level of *p* < 0.05 was utilized to determine statistical significance.

## 3 RESULTS

There were 1178 valid responses. For this analysis, we included only participants who completed the sexual health section (894; 74.9% completion rate). We also excluded participants who have never had sex (*n* = 190).

In the analytic sample (*n* = 704; mean [SD; range] age, 32.5 [13.63; 14–82] years), about one‐third identified as trans women or nonbinary people AFAB. Approximately four‐fifths identified as Pākehā/New Zealand European. Table 1 presents additional demographic characteristics. Appendix S2 presents the genders that our participants were sexually attracted to. Approximately three‐fifths were sexually attracted to trans men (57.2%) or cis men (59.5%) and these participants were grouped together as sexually attracted to men. The largest gender group among our survey participants was non‐binary, and more than one‐fifth were trans men attracted to men or non‐binary AFAB attracted to men.

On average, our participants were at least somewhat certain that they could negotiate protective barrier use with a sexual partner in all situations. Table 3 shows the proportion of participants who were somewhat or completely certain about protective barrier use in each situation. Approximately nine‐tenths were certain that they would use a protective barrier with a new sexual partner, or one who was trans or non‐binary. This was followed by about four‐fifths who felt certain they would refuse sex when a protective barrier was not available or would ask other sexual partners to use one (including someone who truly saw their gender identity, a cisgender partner or when their previous sex together had not involved using a protective barrier). Protective barrier use was lower when participants were drunk or high or when their partner refused to use such protection, with less than three‐fifths reported feeling certain they would use a protective barrier in these circumstances.

**Table 3:** Individual items within the imputed Trans‐Specific Condom/Barrier Negotiation Self‐Efficacy (T‐Barrier) Scale (*N* = 618)

	Mean (SD)	Participants who responded “somewhat certain” and “completely certain” (*n*, %)
I could ask a new sexual partner to use a protective barrier	4.50 (0.94)	551 (89.2)
I could ask a sexual partner I haven't been using protective barriers with to start using them	4.32 (1.02)	519 (84.0)
I could refuse sex when I don't have a protective barrier available	4.32 (1.07)	511 (82.7)
I could get a sexual partner to use a protective barrier, even if I'm drunk or high	3.60 (1.29)	359 (58.1)
I could get a sexual partner to use a protective barrier, even if they don't want to	3.52 (1.31)	342 (55.3)
If a sexual partner truly sees my gender identity, I could ask them to use a protective barrier	4.28 (1.04)	489 (79.1)
I could ask a sexual partner who is cisgender (not trans or non‐binary) to use a protective barrier	4.39 (1.03)	528 (85.4)
I could ask a trans or non‐binary sexual partner to use a protective barrier	4.51 (0.90)	547 (88.5)
T‐Barrier Scale [8–40] (Mean/SD)	33.45 (6.89)	

Abbreviation: SD, standard deviation.

Findings of linear regressions examining factors associated with the T‐Barrier Scale are displayed in Table 4. Differences in the proportion of participants able to negotiate barrier use across gender and sexual attraction groups are outlined in Appendices S3 and S4, respectively. In the multivariate model that adjusted for covariates, trans women not attracted to men (b = –2.40), trans men attracted to men (b = –2.18) and non‐binary AFAB people attracted to men (b = –2.30) had lower average points than trans women attracted to men, of reporting being able to negotiate protective barrier use. Compared to Pākehā/White participants, Asian participants scored 3.46 lower average points in certainty about being able to ask a sexual partner to use protective barriers. See Appendix S5 for the marginal mean of each gender and sexual attraction group.

**Table 4:** Linear regression of T‐Barrier Scale across demographic groups

	Bivariate	Multivariate
	b [95% CI]	b [95% CI]
Age	−0.00 [−0.05 to 0.04]	–
Gender and sexual attraction		
Trans women attracted to men	Ref	Ref
Trans women not attracted to men	−2.28 [−4.26 to −0.31]*	−2.40 [−4.37 to −0.43]*
Trans men attracted to men	−2.26 [−4.00 to −0.52]*	−2.18 [−3.93 to −0.43]*
Trans men not attracted to men	−2.22 [−5.08 to 0.63]	−2.10 [−4.95 to 0.74]
Non‐binary AFAB attracted to men	−2.37 [−4.07 to 3–0.68]**	−2.30 [−4.00 to −0.59]**
Non‐binary AFAB not attracted to men	−1.35 [−3.85 to 1.14]	−1.47 [−3.96 to 1.02]
Non‐binary AMAB attracted to men	−0.84 [−3.15 to 1.47]	−1.03 [−3.34 to 1.28]
Non‐binary AMAB not attracted to men	−1.88 [−5.48 to 1.71]	−2.08 [−5.66 to 1.50]
Prioritized ethnicity groups		
Others including Pākehā/New Zealand European (White)	Ref	Ref
Māori	−0.38 [−1.98 to 1.23]	−0.31 [−1.91 to 1.30]
Pasifika	−1.70 [−4.92 to 1.51]	−2.16 [−5.39 to 1.07]
Asian	−3.84 [−6.70 to −0.98]**	−3.46 [−6.32 to −0.58]*
Income	0.14 [−0.48 to 0.77]	–
Education qualification	0.01 [−0.61 to 0.63]	–
Sex work in the last 12 months	−0.07 [−2.03 to 2.01]	–

Note: *b* refers to the differences in the predicted scores from the respective category to the reference category.

Abbreviations: AFAB, assigned female at birth; AMAB, assigned male at birth; CI, confidence interval.

*p < 0.05; **p < 0.01.

Table 2 presents the proportion of participants who were aware of the efficacy and public funding of PrEP in Aotearoa. About three‐fifths responded that they knew PrEP can reduce the risk of acquiring HIV or that PrEP does not protect against transmission of other STIs. Two‐fifths were aware of the funding of PrEP for males or transpeople who meet other eligibility criteria. A very low proportion of our participants (1.0%) were currently taking PrEP or had previously taken it in the last 6 months. All of these participants responded “I knew that” to each of the three questions about the efficacy of PrEP and its availability for trans people.

We report the differences in proportion across gender and sexual attraction groups for PrEP awareness in Appendices S6 and S7, respectively. Findings of logistic regressions on socio‐demographic characteristics across three variables on PrEP awareness are reported in Table 5. Multivariate models showed trans men attracted to men were significantly more likely than trans women attracted to men to know about PrEP's function in reducing HIV acquisition risk (OR = 2.99), to be aware of PrEP's inability to prevent other STIs (OR = 2.56) and to know about eligibility for publicly funded PrEP in Aotearoa (OR = 2.80). In all multivariate models, participants with higher education qualifications had higher knowledge of PrEP's efficacy and availability.

**Table 5:** Logistic regression of PrEP awareness across demographic groups

	First statement		Second statement		Third statement	
	Bivariate	Multivariate	Bivariate	Multivariate	Bivariate	Multivariate
	OR [95% CI]	OR [95% CI]	OR [95% CI]	OR [95% CI]	OR [95% CI]	OR [95% CI]
Age	0.99 [0.97–1.00]**	0.99 [0.98–1.00]	0.98 [0.97–0.99]**	0.99 [0.97–1.00]	0.99 [0.98–1.00]	–
Gender and sexual attraction						
Trans women attracted to men	Ref		Ref		Ref	Ref
Trans women not attracted to men	0.65 [0.37–1.16]	0.70 [0.39–1.26]	0.51 [0.29–0.92]*	0.53 [0.29–0.98]*	0.59 [0.32–1.09]	0.58 [0.31–1.08]
Trans men attracted to men	3.07 [1.68–5.61]**	2.99 [1.61–5.54]**	2.94 [1.61–5.37]**	2.56 [1.35–4.84]**	2.66 [1.51–4.70]**	2.80 [1.58–4.96]**
Trans men not attracted to men	1.75 [0.75–4.07]	1.76 [0.75–4.14]	1.37 [0.61–3.06]	1.22 [0.53–2.78]	1.62 [0.71–3.72]	1.73 [0.75–4.00]
Non‐binary AFAB attracted to men	1.78 [1.04–3.06]*	1.67 [0.96–2.91]	1.48 [0.86–2.53]	1.26 [0.72–2.23]	0.97 [0.57–1.67]	0.96 [0.55–1.65]
Non‐binary AFAB not attracted to men	2.09 [0.95–4.53]	2.08 [0.95–4.56]	1.37 [0.65–2.87]	1.28 [0.59–2.75]	1.29 [0.62–2.69]	1.31 [0.63–2.75]
Non‐binary AMAB attracted to men	2.03 [0.93–4.41]	1.88 [0.86–4.14]	1.56 [0.74–3.31]	1.34 [0.62–2.90]	1.78 [0.87–3.65]	1.66 [0.80–3.43]
Non‐binary AMAB not attracted to men	0.57 [0.18–1.75]	0.64 [0.20–2.02]	0.51 [0.17–1.60]	0.58 [0.18–1.85]	0.79 [0.25–2.54]	0.83 [0.25–2.70]
Prioritized ethnicity groups	–					
Others including Pākehā/New Zealand European (White)	Ref		Ref		Ref	–
Māori	1.77 [1.00–3.15]	–	1.79 [1.01–3.18]*	1.56 [0.85–2.84]*	1.43 [0.85–2.41]	–
Pasifika	0.69 [0.27–1.77]	–	0.56 [0.22–1.44]	0.55 [0.21–1.48]	0.78 [0.30–2.05]	–
Asian	1.54 [0.54–4.34]	–	0.96 [0.37–2.49]	0.60 [0.22–1.60]	1.63 [0.68–3.95]	–
Income	1.09 [0.90–1.33]	–	1.05 [0.87–1.27]	–	1.19 [0.99–1.44]	–
Education qualification	1.33 [1.09–1.63]**	1.41 [1.14–1.75]**	1.39 [1.14–1.69]**	1.50 [1.21–1.86]**	1.36 [1.12–1.65]**	1.41 [1.16–1.73]**
Sex work in the last 12 months	1.44 [0.70–2.93]	–	1.86 [0.87–4.00]		1.46 [0.77–2.79]	–

*Note*. First—PrEP (pre‐exposure prophylaxis) is a pill that, if taken every day by someone who is HIV negative, significantly decreases their risk of acquiring HIV.

Second—If taken correctly, PrEP significantly reduces the risk of acquiring HIV but it does not prevent the transmission of other STIs like gonorrhoea and syphilis.

Third—PrEP is now publicly funded in New Zealand, if you are “male or transgender” and meet other eligibility criteria.

Abbreviations: AFAB, assigned female at birth; AMAB, assigned male at birth; CI, confidence interval; OR, odds ration; PrEP, pre‐exposure prophylaxis.

*p < 0.05; **p < 0.01.

## 4 DISCUSSION

These data are the first from Aotearoa measuring the ability of a diverse range of trans people to access two important HIV prevention strategies, through negotiating barrier protection and building awareness of PrEP's efficacy and availability. Current research on HIV prevention among trans people has primarily focused on trans women [22]. Any automatic generalization of findings from these studies to the larger population of trans people should be made with caution as studies have documented variability in health experiences for trans women, trans men and non‐binary people [2, 17, 23].

In our study, trans women who were attracted to men were more likely to report certainty about using protective barriers compared to trans women not attracted to men. Previous studies on the T‐Barrier Scale have produced mixed findings on gendered differences. For instance, Trans PULSE did not detect significant gendered differences between AFAB and AMAB groups [18] and a Brazilian study reported a higher perceived ability to negotiate protective barriers (e.g. condoms) among trans men than trans women [21]. Our study asserted the importance of examining the intersection of gender and sexual attraction, as we only found significant gendered differences among those attracted to men: trans women had higher certainty around negotiating protective barrier use than trans men and non‐binary AFAB people. Future research is required to examine correlates of T‐Barrier Scale (e.g. self‐esteem, experiences of stigma and discrimination, and the types of protective barrier used) for trans women, trans men and nonbinary people disaggregated by sexual attraction.

In this study, Asian trans participants had a lower ability to negotiate protective barrier use. Earlier published Counting Ourselves data also showed they were more likely to have been rejected by a family member because they were trans or non‐binary [3, 24]. In contrast, a previous US study found young trans women who reported having parental support consistently practiced safe sex [25]. Other Aotearoa research has identified difficulties negotiating protective barrier use with a sexual partner can also be due to power asymmetry (including interpersonal differences in language, age, sexual experience or openness about one's sexuality) [14]. Our study points towards a need to facilitate sexual health equity for Asian trans people. Sexual health services can play an important role by providing language support, promoting culturally safe care and addressing institutional racism [26, 27].

PrEP should benefit those most at risk, not just those most able to navigate healthcare systems [15]. In line with previous studies [28, 29], our trans participants with a higher level of education qualification had higher awareness of PrEP. Previous studies comparing trans people's PrEP awareness based on sexual attraction have mostly focused on trans women and non‐binary people AMAB [29, 30]. Our study provided more nuanced findings by exploring the differences in PrEP awareness for trans women, trans men and non‐binary people who are sexually attracted to men. Among those sexually attracted to men, we found lower levels of PrEP awareness among trans women compared to trans men. Our finding on gender differences is similar to the US TransPoP survey that found a lower level of PrEP familiarity among trans people AMAB than those AFAB [31].

Trans and non‐binary people in Aotearoa commonly access gender‐affirming healthcare through doctors working as general practitioners (GPs) in primary care settings [3]. This reflects GPs’ role in prescribing subsidized hormones and referring trans people to the limited number and range of surgeries available through public hospitals. Yet, earlier Counting Ourselves findings revealed that almost half of our participants (48%) were uncomfortable or very uncomfortable discussing being trans or non‐binary with their GP, rising to over two‐thirds (68%) with a new GP [3]. Increasing PrEP uptake among trans people in Aotearoa is likely to require upskilling GPs about the newly released PrEP guidelines [12], alongside an already identified need to improve primary healthcare providers’ competency in delivering general and gender‐affirming care to trans people [32].

There are several limitations to consider in this study. Counting Ourselves utilized convenience sampling which led to over‐recruitment of participants, who were younger, from urban regions and more connected to trans community organizations. However, this method resulted in a sample size that was many times larger than other national trans surveys relative to the overall population [17]. Similar to overseas nationwide community‐based studies [17], our non‐binary sample contained a smaller proportion of AMAB participants (24.8%). Only 2.7% (*n* = 19) of our overall sample were non‐binary AMAB attracted to men. Considering our novel finding on the variability in protective barrier use and PrEP awareness across gender and sexual attraction groups, we recommend future studies recruit a large representative sample of trans people to better understand these nuances.

We used the sexual attraction question from Counting Ourselves, as this measured participants’ current sexual attraction to one or more gender groups. The survey also asked participants “who they had ever sex with,” with the same range of response options. As the sexual behaviour question encompasses lifetime sexual experiences, some of these would have been when participants identified as cisgender. Our survey questionnaire did not include a sexual identity question. There is a need for more detailed survey questions that enable trans people to describe the diverse complexity of sexual orientation, attraction and behaviour across time and gender transitions.

## 5 CONCLUSIONS

Participants attracted to men have a higher potential need for PrEP. This group was more likely to report PrEP awareness and that they could negotiate protective barrier usage. However, among participants attracted to men, we found trans women had less PrEP awareness than trans men, and awareness was lower for those with a lower educational qualification. Asian participants were less certain they could negotiate the use of protective barriers. More trans‐competent, accessible sexual health education, drawing on the newly released PrEP guidelines, is needed to promote the benefits of PrEP in the Aotearoa HIV epidemic context, particularly for trans women. Improving the use of PrEP is likely to require upskilling primary healthcare providers to improve their knowledge and cultural competency around supporting all trans people.

## COMPETING INTERESTS

The authors declare that they have no competing interests.

## AUTHORS’ CONTRIBUTIONS

All authors were involved in the development of the study and JLB, KKHT and PJS wrote the manuscript. KKHT and RMB analysed the data. JFV provided essential input on the analysis and manuscript. All authors critically reviewed and edited the manuscript.

## FUNDING

This work was supported by the Health Research Council of New Zealand [JFV, 17/587], the Rule Foundation and the University of Waikato. PS's involvement was supported by the NZ AIDS Foundation Fellowship at the University of Auckland.

## Supporting information


**Appendix 1**. Missing Value Analysis of T‐Barrier Scale.
**Appendix 2**. Sexual Attraction towards Gender Groups (*n* = 699).
**Appendix 3**. Proportions of Participants who were “Somewhat certain” and “Completely certain” for the Imputed T‐Barrier Scale across Gender Groups.
**Appendix 4**. Proportions of Participants who were “Somewhat certain” and “Completely certain” for the 8‐item T‐Barrier Scale across Sexual Attraction Groups.
**Appendix 5**. Estimated Marginal Mean for T‐Barrier Scale for each Gender and Sexual Attraction Group.
**Appendix 6**. Proportions of Participants who Responded “I knew that” about PrEP Information across Gender Groups.
**Appendix 7**. Proportions of Participants who Responded “I knew that” about PrEP Information across Sexual Attraction Groups.

## Data Availability

The data that support the findings of this study are available on request from the corresponding author. The data are not publicly available due to privacy or ethical restrictions.
