# Supplementary material for: PrEP awareness and protective barrier negotiation among transgender people attracted to men in Aotearoa New Zealand
Source: J Int AIDS Soc. 2022 Oct 12;25(Suppl 5):e25980. doi: 10.1002/jia2.25980 (PMC9557014; doi:10.1002/jia2.25980)
Supplement: Supplementary file 1 — Appendix 1. Missing Value Analysis of T‐Barrier Scale. Appendix 2. Sexual Attraction towards Gender Groups (n = 699). Appendix 3. Proportions of Participants who were “Somewhat certain” and “Completely certain” for the Imputed T‐Barrier Scale across Gender Groups. Appendix 4. Proportions of Participants who were “Somewhat certain” and “Completely certain” for the 8‐item T‐Barrier Scale across Sexual Attraction Groups. Appendix 5. Estimated Marginal Mean for T‐Barrier Scale for each Gender and Sexual Attraction Group. Appendix 6. Proportions of Participants who Responded “I knew that” about PrEP Information across Gender Groups. Appendix 7. Proportions of Participants who Responded “I knew that” about PrEP Information across Sexual Attraction Groups. [file JIA2-25-e25980-s001.docx]

**Appendices**

| Appendix 1. Missing Value Analysis of T-Barrier Scale | | |
| --- | --- | --- |
|  | Proportion of participants selected “This does not apply” | Percentage of missing values including “This does not apply” |
| I could ask a new sexual partner to use a protective barrier | 85 (12.3) | 85 (12.3) |
| I could ask a sexual partner I haven't been using protective barriers with to start using them | 106 (15.4) | 106 (15.4) |
| I could refuse sex when I don't have a protective barrier available | 73 (10.6) | 74 (10.7) |
| I could get a sexual partner to use a protective barrier, even if I'm drunk or high | 147 (21.6) | 155 (22.5) |
| I could get a sexual partner to use a protective barrier, even if they don't want to | 108 (15.8) | 114 (16.5) |
| If a sexual partner truly sees my gender identity, I could ask them to use a protective barrier | 126 (18.7) | 140 (20.3) |
| I could ask a sexual partner who is cisgender (not trans or non-binary) to use a protective barrier | 84 (12.3) | 92 (13.4) |
| I could ask a trans or non-binary sexual partner to use a protective barrier | 109 (16.1) | 121 (17.6) |

| Appendix 2. Sexual Attraction towards Gender Groups (*n* = 699) | |
| --- | --- |
|  | n (%) |
| Trans men | 399 (57.2) |
| Cis men | 416 (59.5) |
| Trans women | 458 (65.6) |
| Cis women | 566 (81.0) |
| Genderqueer or nonbinary people | 470 (66.8) |
| None of the above | 45 (6.4) |
| Others | 42 (6.0) |

Participants were those who have had sex. Others including those that we could not recoded into existing categories such as polyamorous, unsure, and undecided.

| Appendix 3. Proportions of Participants who were “Somewhat certain” and “Completely certain” for the Imputed T-Barrier Scale across Gender Groups | | | | | |
| --- | --- | --- | --- | --- | --- |
|  | Trans women  n (%) | Trans men  n (%) | Non-binary AFAB n (%) | Non-binary AMAB n (%) | Chi-square results |
| I could ask a new sexual partner to use a protective barrier (*n*=603) | 171 (91.4) | 146 (87.4) | 174 (88.3) | 59 (89.4) | **ꭓ^2^(6) = 15.68, *p* = .016** |
| I could ask a sexual partner I haven't been using protective barriers with to start using them (*n*=582) | 160 (85.6) | 136 (81.4) | 164 (83.2) | 58 (87.9) | ꭓ^2^(6) = 2.38, *p* = .881 |
| I could refuse sex when I don't have a protective barrier available (*n*=614) | 157 (84.0) | 139 (83.2) | 168 (85.3) | 55 (83.3) | ꭓ^2^(6) = 2.15, *p* = .905 |
| I could get a sexual partner to use a protective barrier, even if I'm drunk or high (*n*=533) | 112 (59.9) | 94 (56.3) | 113 (57.4) | 44 (66.7) | ꭓ^2^(6) = 2.87, *p* = .826 |
| I could get a sexual partner to use a protective barrier, even if they don't want to (*n*=574) | 113 (60.4) | 85 (50.9) | 105 (53.3) | 38 (57.6) | ꭓ^2^(6) = 7.26, *p* = .298 |
| If a sexual partner truly sees my gender identity, I could ask them to use a protective barrier (*n*=548) | 155 (82.9) | 127 (76.0) | 152 (77.2) | 54 (81.8) | ꭓ^2^(6) = 3.95, *p* = .683 |
| I could ask a sexual partner who is cisgender (not trans or non-binary) to use a protective barrier (*n*=596) | 168 (89.8) | 137 (82.0) | 163 (82.7) | 59 (89.4) | ꭓ^2^(6) = 10.12, *p* = .120 |
| I could ask a trans or non-binary sexual partner to use a protective barrier (*n*=567) | 165 (88.2) | 148 (88.6) | 174 (88.3) | 59 (89.4) | ꭓ^2^(6) = 0.14, *p* = 1.000 |

Note. Pearson chi-square analyses were carried out in three response categories “Certain”, “Neither certain nor uncertain” and “Uncertain”.

One nonbinary participant did not report sex assigned at birth and was excluded from this analysis.

| Appendix 4. Proportions of Participants who were “Somewhat certain” and “Completely certain” for the 8-item T-Barrier Scale across Sexual Attraction Groups | | | | | |
| --- | --- | --- | --- | --- | --- |
|  | Trans men  *n* (%) | Trans women  *n* (%) | Cis men  *n* (%) | Cis women  *n* (%) | Genderqueer or nonbinary people  *n* (%) |
| I could ask a new sexual partner to use a protective barrier | 343 (90.7)  ꭓ^2^(2) = 3.64, *p* = .162 | 398 (91.1)  ꭓ^2^(2) = 5.43, *p* = .066 | **365 (91.9)**  **ꭓ^2^(2) = 8.55, *p* = .014** | 461 (89.0)  ꭓ^2^(2) = 0.33, *p* = .847 | 402 (89.8)  ꭓ^2^(2) = 1.34, *p* = .512 |
| I could ask a sexual partner I haven't been using protective barriers with to start using them | 325 (86.0)  ꭓ^2^(2) = 2.90, *p* = .234 | 377 (86.3)  ꭓ^2^(2) = 5.69, *p* = .058 | **348 (87.7)**  **ꭓ^2^(2) = 10.80, *p* = .005** | 431 (83.2)  ꭓ^2^(2) = 1.92, *p* = .383 | 379 (84.8)  ꭓ^2^(2) = 2.55, *p* = .280 |
| I could refuse sex when I don't have a protective barrier available | 321 (84.9)  ꭓ^2^(2) = 0.82, *p* = .663 | 368 (84.2)  ꭓ^2^(2) = 0.22, *p* = .897 | 339 (85.4)  ꭓ^2^(2) = 1.41, *p* = .493 | 430 (83.0)  ꭓ^2^(2) = 2.93, *p* = .231 | 372 (83.2)  ꭓ^2^(2) = 0.92, *p* = .631 |
| I could get a sexual partner to use a protective barrier, even if I'm drunk or high | 222 (58.7)  ꭓ^2^(2) = 0.52, *p* = .769 | 253 (57.9)  ꭓ^2^(2) = 0.93, *p* = .629 | 239 (60.2)  ꭓ^2^(2) = 1.21, *p* = .545 | 295 (56.9)  ꭓ^2^(1) = 5.57, *p* = .062 | 254 (56.8)  ꭓ^2^(2) = 3.56, *p* = .169 |
| I could get a sexual partner to use a protective barrier, even if they don't want to | 201 (53.2)  ꭓ^2^(2) = 2.28, *p* = .321 | 236 (54.0)  ꭓ^2^(2) = 1.31, *p* = .520 | 225 (56.7)  ꭓ^2^(2) = 1.09, *p* = .580 | **269 (51.9)**  **ꭓ^2^(2) = 15.99, *p* < .001** | **231 (51.7)**  **ꭓ^2^(2) = 9.51, *p* = .009** |
| If a sexual partner truly sees my gender identity, I could ask them to use a protective barrier) | 301 (79.6)  ꭓ^2^(2) = 0.42, *p* = .810 | 353 (80.8)  ꭓ^2^(2) = 2.43, *p* = .296 | 325 (81.9)  ꭓ^2^(2) = 4.79, *p* = .091 | **401 (77.4)**  **ꭓ^2^(2) = 6.83, *p* = .033** | 352 (78.7)  ꭓ^2^(2) = 0.24, *p* = .885 |
| I could ask a sexual partner who is cisgender (not trans or non-binary) to use a protective barrier | 325 (86.0)  ꭓ^2^(2) = 0.17, *p* = .918 | 377 (86.3)  ꭓ^2^(2) = 1.84, *p* = .399 | **355 (89.4)**  **ꭓ^2^(2) = 13.91, *p* < .001** | 436 (84.2)  ꭓ^2^(2) = 5.03, *p* = .081 | 382 (85.5)  ꭓ^2^(2) = 0.52, *p* = .773 |
| I could ask a trans or non-binary sexual partner to use a protective barrier | 343 (90.7)  ꭓ^2^(2) = 4.63, *p* = .099 | **398 (91.1)**  **ꭓ^2^(2) = 9.43, *p* = .009** | **367 (92.4)**  **ꭓ^2^(2) = 16.81, *p* <.001** | 454 (87.6)  ꭓ^2^(2) = 3.42, *p* = .181 | 401 (89.7)  ꭓ^2^(2) = 2.17, *p* = .338 |

Note. Pearson chi-square analyses were carried out in three response categories “Certain”, “Neither certain nor uncertain” and “Uncertain”.

| Appendix 5. Estimated Marginal Mean for T-Barrier Scale for each Gender and Sexual Attraction Group | |
| --- | --- |
|  | Mean [95% Confidence Interval] |
| Trans women attracted to men | 33.93 [32.31-35.55] |
| Trans women not attracted to men | 31.53 [29.71-33.35] |
| Trans men attracted to men | 31.75 [30.27-33.23] |
| Trans men not attracted to men | 31.83 [29.15-34.51] |
| Non-binary AFAB attracted to men | 31.63 [30.31-33.06] |
| Non-binary AFAB not attracted to men | 32.46 [30.08-34.83] |
| Non-binary AMAB attracted to men | 32.90 [30.70-35.09] |
| Non-binary AMAB not attracted to men | 31.85 [28.33-35.36] |

Mean values were adjusted for ethnicity in generalised regression analyses.

| Appendix 6. Proportions of Participants who Responded “I knew that” about PrEP Information across Gender Groups | | | | | |
| --- | --- | --- | --- | --- | --- |
|  | Trans women  *n* (%) | Trans men  *n* (%) | Non-binary AFAB  *n* (%) | Non-binary AMAB  *n* (%) | Chi-square results |
| PrEP (Pre-Exposure Prophylaxis) is a pill that, if taken every day by someone who is HIV-negative, significantly decreases their risk of acquiring HIV | 119 (46.5) | 151 (59.9) | 160 (56.5) | 43 (52.4) | ꭓ^2^(6) = 11.55, *p* = .073 |
| If taken correctly, PrEP significantly reduces the risk of acquiring HIV but it does not prevent the transmission of other STIs like gonorrhoea and syphilis | 118 (46.3) | 156 (62.2) | 159 (56.2) | 43 (53.1) | **ꭓ^2^(6) = 14.10, *p* = .029** |
| PrEP is now publicly funded in New Zealand, if you are “male or transgender” and meet other eligibility criteria | 73 (28.7) | 111 (44.2) | 93 (32.9) | 32 (39.5) | **ꭓ^2^(6) = 16.69, *p* = .010** |

Note. Pearson chi-square analyses were carried out in three response categories “I knew that”, “I wasn’t sure”, and “I didn’t know that”

| Appendix 7. Proportions of Participants who Responded “I knew that” about PrEP Information across Sexual Attraction Groups | | | | | |
| --- | --- | --- | --- | --- | --- |
|  | Trans men  *n* (%) | Trans women  *n* (%) | Cis men  *n* (%) | Cis women *n* (%) | Genderqueer or nonbinary people  *n* (%) |
| PrEP (Pre-Exposure Prophylaxis) is a pill that, if taken every day by someone who is HIV-negative, significantly decreases their risk of acquiring HIV | **295 (60.5)**  **ꭓ^2^(2) = 16.69, *p* <.001** | **326 (59.4)**  **ꭓ^2^(2) = 14.75, *p* <.001** | **304 (60.2)**  **ꭓ^2^(2) = 15.49, *p* <.001** | 377 (56.4)  ꭓ^2^(2) = 3.36, *p* = .186 | **340 (60.8)**  **ꭓ^2^(2) = 25.58, *p* <.001** |
| If taken correctly, PrEP significantly reduces the risk of acquiring HIV but it does not prevent the transmission of other STIs like gonorrhoea and syphilis | **296 (60.8)**  **ꭓ^2^(2) = 16.83, *p* <.001** | **327 (59.8)**  **ꭓ^2^(2) = 15.51, *p* <.001** | **302 (60.0)**  **ꭓ^2^(1) = 11.92, *p* = .003** | 377 (56.6)  ꭓ^2^(2) = 5.56, *p* = .062 | **338 (60.6)**  **ꭓ^2^(2) = 23.14, *p* < .001** |
| PrEP is now publicly funded in New Zealand, if you are “male or transgender” and meet other eligibility criteria | **194 (39.8)**  **ꭓ^2^(2) = 9.82, *p* = .007** | 207 (37.9)  ꭓ^2^(2) = 5.06, *p* = .080 | **196 (39.0)**  **ꭓ^2^(2) = 6.14, *p* = .046** | 242 (36.3)  ꭓ^2^(2) = 2.16, *p* = .340 | **221 (39.6)**  **ꭓ^2^(2) = 12.01, *p* = .002** |

Note. Pearson chi-square analyses were carried out in three response categories “I knew that”, “I wasn’t sure”, and “I didn’t know that”
